# The Impact of Wireless Emergency Alerts on a Floating Population in Seoul, South Korea: Panel Data Analysis

**DOI:** 10.2196/43554

**Published:** 2024-03-25

**Authors:** Sungwook Yoon, Hyungsoo Lim, Sungho Park

**Affiliations:** 1 Korea Information Society Development Institute Jincheon Republic of Korea; 2 Department of Information Systems, Business Statistics, and Operations Management School of Business and Management Hong Kong University of Science and Technology Clear Water Bay China (Hong Kong); 3 Business School Seoul National University Seoul Republic of Korea

**Keywords:** COVID-19, empirical identification, floating population, social distancing, wireless emergency alert

## Abstract

**Background:**

Wireless emergency alerts (WEAs), which deliver disaster information directly to individuals’ mobile phones, have been widely used to provide information related to COVID-19 and to encourage compliance with social distancing guidelines during the COVID-19 pandemic. The floating population refers to the number of people temporarily staying in a specific area, and this demographic data can be a useful indicator to understand the level of social distancing people are complying with during the COVID-19 pandemic.

**Objective:**

This study aimed to empirically analyze the impact of WEAs on the floating population where WEAs were transmitted in the early stages of the COVID-19 pandemic. As most WEA messages focus on compliance with the government’s social distancing guidelines, one of the goals of transmitting WEAs during the COVID-19 pandemic is to control the floating population at an appropriate level.

**Methods:**

We investigated the empirical impact of WEAs on the floating population across 25 districts in Seoul by estimating a panel regression model at the district-hour level with a series of fixed effects. The main independent variables were the number of instant WEAs, the daily cumulative number of WEAs, the total cumulative number of WEAs, and information extracted from WEAs by natural language processing at the district-hour level. The data set provided a highly informative empirical setting as WEAs were sent by different local governments with various identifiable district-hour–level data.

**Results:**

The estimates of the impact of WEAs on the floating population were significantly negative (–0.013, *P*=.02 to –0.014, *P*=.01) across all specifications, implying that an additional WEA issuance reduced the floating population by 1.3% (=100(1–e^–0.013^)) to 1.4% (=100(1–e^–0.014^)). Although the coefficients of *DCN* (the daily cumulative number of WEAs) were also negative (–0.0034, *P*=.34 to –0.0052, *P*=.15) across all models, they were not significant. The impact of WEAs on the floating population doubled (–0.025, *P*=.02 to –0.033, *P*=.005) when the first 82 days of observations were used as subsamples to reduce the possibility of people blocking WEAs.

**Conclusions:**

Our results suggest that issuing WEAs and distributing information related to COVID-19 to a specific district was associated with a decrease in the floating population of that district. Furthermore, among the various types of information in the WEAs, location information was the only significant type of information that was related to a decrease in the floating population. This study makes important contributions. First, this study measured the impact of WEAs in a highly informative empirical setting. Second, this study adds to the existing literature on the mechanisms by which WEAs can affect public response. Lastly, this study has important implications for making optimal WEAs and suggests that location information should be included.

## Introduction

The frequency of disasters and the scale of the damage have increased dramatically over the decades. Natural disasters related to climate, weather, or water hazards have increased more than 5-fold over the past 50 years. Such incidents have resulted in approximately 115 casualties and US $202 million in damage per day [1]. Likewise, man-made disasters, including criminal attacks (eg, shooter incidents and terrorist attacks), human-based technological incidents (eg, building or bridge collapses), and events related to the manufacture, transportation, storage, and use of hazardous materials, have also increased significantly over the past 50 years. Moreover, the number of occurrences was estimated to have increased 6-fold or more, whereas the amount of damage increased by approximately 189 times [2].

Along with a significant number of disasters, the importance of wireless emergency alerts (WEAs) to rapidly deliver disaster information is emphasized. Many countries, including the United States, Japan, and South Korea, have adopted WEAs as a disaster information delivery system channel. This is because WEAs operate as a cell broadcast service (CBS) system that can send messages to multiple mobile phone users in a defined area simultaneously. Information is delivered directly to an individual’s cell phone; hence, it can be easily accessed through an individual’s mobile phone without the hassle of searching.

WEAs have been actively used to prevent the spread of COVID-19 since early 2020 [3]. In the United States, Utah and Massachusetts have issued WEAs reminding the populace of safety rules related to COVID-19 [4,5]. In New Zealand, WEAs were sent to mobile phone users across the country in the name of the National Emergency Management Agency [6]. Taiwan also used a cell-based mass texting system known as the Public Warning CBS to share information on COVID-19 [7].

One of the most efficient ways to respond to a disaster situation is to evacuate people from the danger zone to a safer location. Therefore, it is necessary to monitor changes in the flow of public movement, including the floating population, among the various public responses that occur during such disasters. Along with the growing frequency of disasters, there is increasing demand for countries or local governments to examine the impact of WEAs, as it could be one of the solutions to prevent severe damage from emergent events. WEAs are an information delivery channel optimized for this purpose, enabling people in a specific area to receive disaster notifications on their cell phones and prompting them to evacuate. Therefore, analyzing the relationship between the issuance of WEAs and the size of the floating population in a given area is the most intuitive and effective way to measure the policy performance of sending WEAs, which provides important implications for developing future policies. However, little is known about the impact of WEAs on people’s behavior because granular data sets of the focal outcome variable at various regions and short-time levels are unlikely to be available (eg, the real-time population at a specific time in a particular region). Only a few studies have examined the impact of WEAs, and they focused on either the impact of a single region [8] or simply compared the delivering and nondelivering regions [3].

We empirically investigated the associations between WEAs and the floating population in 25 districts in Seoul, the largest metropolis in South Korea, in the early stages of the COVID-19 pandemic for 13 months, from January 2020 to January 2021. The floating population denotes the transient influx of individuals within a defined area, and this metric serves as a pivotal indicator, offering perspectives on the adherence to social distancing protocols during the COVID-19 pandemic. As most WEA messages focus on compliance with the government’s social distancing guidelines, we can safely say that the goal of transmitting WEAs during the COVID-19 pandemic is to control the floating population at an appropriate level.

## Methods

### Data Set

In May 2005, the South Korean government established a nationwide WEA transmission system for the first time in the world, and since 2013, the WEA reception function has been mandatory for all mobile phones. WEAs operate as a CBS system, a method of simultaneously sending messages to multiple mobile phone users in a defined area. CBS is a one-to-many geo-targeted and geo-fenced messaging service, unlike SMS.

Since the establishment of the WEA transmission system, only the central government has had the authority to send WEAs, but this authority was extended to local governments in 2019 with an amendment to the relevant law. Since then, local governments have sent WEAs related to natural disasters, such as storms, floods, conflagrations, and other issues in their jurisdictions. WEA sending standards and the composition of contents are the subjects of the authority’s autonomy. Unlike WEAs sent by the central government, those from the local governments are sent only to their jurisdictional areas.

We have collected 6442 WEAs sent by each local government in the designated district (Gu—administrative district in Seoul, and there are 25 of them) in Seoul for 13 months from January 2020 to January 2021. Detailed WEAs were extracted from the “Public Data Portal” built by the Ministry of the Interior and Safety of the South Korean government. The empirical period covers the early stage of the COVID-19 pandemic in South Korea.

To investigate what kind of information should be included in the WEAs, we extracted information from the WEAs using natural language processing. The first information extracted from WEAs was whether or not the WEAs contained location information. The second indicated whether the WEAs had up-to-date information. The third indicated whether the WEAs had information on hand (eg, number of confirmed cases). The final one indicated whether the WEAs had disease information (eg, detailed information on COVID-19).

For the dependent variable, we used the estimated floating population provided by SK Telecom, which has the largest number of subscribers in South Korea, at the district-hour level during the same period. We summarized the materials that aid in the understanding of our dependent and independent variables in Multimedia Appendix 1.

### Empirical Model

We specified our empirical model at the region-hour level as follows:



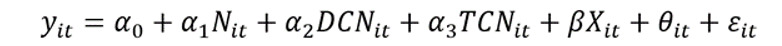



where 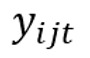 denotes the dependent variable in district *i* at time *t*; *N_it_* indicates the number of WEAs in district *i* at time *t*; *DCN_it_* indicates the daily cumulative number of WEAs in district *i* until time *t* (excluding the number of WEAs at time *t*); *TCN_it_* indicates the total cumulative number of WEAs in district *i* until time *t* since January 1, 2020; *X_it_* indicates the information extracted from WEAs with natural language processing; and 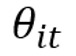 indicates the group of fixed effects that includes district-fixed effects, time-fixed effects, and joint district and time-fixed effects. To control the effect of unobservable factors (eg, daily work commute), we included these granular regions, weekday, and hour-fixed effects. This specification alleviated the concern about controlling unobservable factors that could affect the floating population.

The dependent variable was the floating population at time *t* in district *i*. We specified continuous variables in logarithms since they have a skewed distribution (eg, 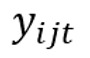 and *TCN_it_*). As appropriate, we added 1 to the variables to avoid logarithms of 0. Table 1 shows the distribution of *N* and *DCN* during the day. The first column represents 24 hours. For instance, 0 indicates a period from midnight to 1 AM. The second and third columns represent the average *N* (in gray) and *DCN* (in light gray) across 25 districts. As shown in Table 1, *DCN* increased over time during a day as *N* accumulated. On average, people received 0.65 WEAs daily, and the peak time was 6 PM. Tables 2 and 3 report the summary statistics and correlations of the variables. Most of the correlations are significant (*P*<.001) except for a correlation between floating population and number of WEAs with order information, as shown in Table 3. The correlations between each variable were mostly at a modest level, and considering our sample size (the number of observations in the analysis is 236,775), we concluded that each variable had different variations. We used the ordinary least squares method to estimate the unknown parameters of our empirical model.

Various types of information were sent through WEAs, and natural language processing techniques were used to extract and identify them. Sentences in WEAs were stemmed through a morphology analyzer named “Mecab” and “Okt tagger” in KoNLPY, an open-source Korean language processing package. After classifying common nouns, proper nouns, adjectives, and verbs through the morphology analyzer, informative words were extracted by specifying a list for the exclusion of stopwords and out-of-vocabulary words in advance. The type of information included in the WEAs was identified by classifying the extracted words according to a predetermined semantic standard (named entity recognition; NER). We used the NER application programming interface (API) of the AI API/Data service provided by the Electronics and Telecommunications Research Institute, Korea; this API is known to have a high performance of 89.4% in Korean NER [9].

**Table 1:** N (number of wireless emergency alerts [WEAs]) and DCN (daily cumulative number of WEAs) at hour level for WEAs issued in 25 districts of Seoul between January 2020 and January 2021.

Hour	*N*	*DCN*
0	0.000	0.000
1	0.000	0.000
2	0.000	0.000
3	0.000	0.000
4	0.000	0.000
5	0.000	0.000
6	0.001	0.000
7	0.002	0.001
8	0.004	0.004
9	0.030	0.008
10	0.058	0.038
11	0.052	0.097
12	0.018	0.149
13	0.036	0.166
14	0.042	0.203
15	0.047	0.244
16	0.056	0.291
17	0.088	0.347
18	0.122	0.435
19	0.047	0.557
20	0.025	0.604
21	0.016	0.629
22	0.003	0.645
23	0.000	0.649

**Table 2:** Descriptive statistics of the floating population and wireless emergency alerts (WEAs) across 25 districts in Seoul between January 2020 and January 2021.

Variables	Median	Mean	Minimum	Maximum
1. Floating population	319,000	327,382	94,020	1,044,150
2. Number of WEAs (*N*^a^)	0	0.027	0	4
3. Number of daily cumulative WEAs (*DCN*^b^)	0	0.211	0	8
4. Number of total cumulative WEAs (*TCN*^c^)	54	84.210	0	495
5. Number of WEAs with location information	0	0.017	0	9
6. Number of WEAs with date information	0	0.007	0	8
7. Number of WEAs with order information	0	0.040	0	11
8. Number of WEAs with disease information	0	0.005	0	4

^a^*N*: number of WEAs.

^b^*DCN*: the daily cumulative number of WEAs in a specific district.

^c^*TCN*: the total cumulative number of WEAs in a specific district since January 1, 2020, the beginning of the observation period.

**Table 3:** Correlation matrix of the floating population and wireless emergency alerts (WEAs) across 25 districts in Seoul between January 2020 and January 2021.

Correlation^a^	(1)	(2)	(3)	(4)	(5)	(6)	(7)	(8)
(1) Log (floating population)	1.000	0.016	0.028	0.153	0.005^b^	0.029	0.001^c^	0.015
(2) Number of WEAs (*N*^d^)	0.016	1.000	0.042	0.106	0.564	0.437	0.805	0.400
(3) Number of daily cumulative WEAs (*DCN*^e^)	0.028	0.042	1.000	0.265	0.014	0.025	0.028	0.005^b^
(4) Number of total cumulative WEAs (*TCN*^f^)	0.153	0.106	0.265	1.000	0.033	0.042	0.103	0.023
(5) Number of WEAs with location information	0.005^b^	0.564	0.014	0.033	1.000	0.216	0.469	0.173
(6) Number of WEAs with date information	0.029	0.437	0.025	0.042	0.216	1.000	0.285	0.195
(7) Number of WEAs with order information	0.001^c^	0.805	0.028	0.103	0.469	0.285	1.000	0.284
(8) Number of WEAs with disease information	0.015	0.400	0.005^b^	0.023	0.173	0.195	0.284	1.000

^a^All correlations are significant (*P*<.001) if there is no note.

^b^Correlation is significant (*P*=.01).

^c^Correlation is insignificant (*P*=.65).

^d^*N*: number of WEAs.

^e^*DCN*: the daily cumulative number of WEAs in a specific district.

^f^*TCN*: the total cumulative number of WEAs in a specific district since January 1, 2020, the beginning of the observation period.

### Ethical Considerations

To compile the data set used in this study, publicly available information was collected through the Ministry of the Interior and Safety of the Republic of Korea. We received confirmation of exemption from the Public Institutional Bioethics Committee designated by the Ministry of Health and Welfare of the Republic of Korea (P01-202309-01-043).

## Results

Parameter estimates of our empirical model are shown in Table 4. We included district, weekday, hour, and joint district-hour fixed effects in all regressions.

As shown in the first row of Table 4 (*N*), the estimates were significantly negative across all models, implying that when people received WEAs, the floating population decreased. Based on the estimate in column 2, one increase in WEA was related to a reduction of the floating population by 1.3% (=100(1 – e^–0.013^)). Other specifications yielded similar results, ranging from 1.3% (=100(1 – e^–0.013^)) to 1.4% (=100(1 – e^–0.014^)). Although the coefficients of *DCN* were also negative across all models and related to a reduction of the floating population from 0.34% (=100(1 – e^–0.0034^)) to 0.52% (=100(1 – e^–0.0052^)) (), they were insignificant. To check the impact of cumulative WEAs, we incorporated the *TCN* into the main specification, as shown in column 2 of Table 4. The results show that cumulative WEAs were also negatively correlated with the floating population. We replaced *TCN* with *TCC* (total number of confirmed cases of COVID-19) and *Day* (daily time trend) to check the robustness and summarize the results in columns 3 and 4 of Table 4, respectively. We used the variables related to *TCN*, *TCC*, and *Day* separately due to their high correlation (0.963 between *log (TCN)* and *log (TCC)*; 0.902 between *log (TCN)* and *log (Day)*; and 0.899 between *log (TCC)* and *log (Day)*). As shown in columns 3 and 4, *TCC* and *Day* had negative impacts on the floating population, like *TCN*.

We conducted several robustness checks with subsamples and different specifications. The results of the robustness checks were consistent with our main findings. Subsequently, we performed 2 falsification tests, and we found that our results did not hold when using incorrect variables in terms of district and timing. Detailed information and results of the robustness checks and falsification tests are summarized in Multimedia Appendix 2.

**Table 4:** Parameter estimates of the main empirical model: panel regression model using wireless emergency alerts (WEAs) and the floating population at the district-hour level with a series of fixed effects.

	(1) Base	(2) With *TCN*^a^	(3) With *TCC*^b^	(4) With day
*N* ^c^	−0.014 (0.0053; *P*=.01)	−0.013 (0.0050; *P*=.02)	−0.013 (0.0048; *P*=.01)	−0.013 (0.0050; *P*=.02)
*DCN* ^d^	−0.0052	−0.0034	−0.0039	−0.0037
	(0.0035; *P*=.15)	(0.0035; *P*=.34)	(0.0034; *P*=.26)	(0.0035; *P*=.30)
*Log (TCN)*	N/A^e^	−0.0019 (0.0005; *P*=.002)	N/A	N/A
*Log (TCC)*	N/A	N/A	−0.0012 (0.0006; *P*=.06)	N/A
*Log (Day)*	N/A	N/A	N/A	−0.0033 (0.0010; *P*=.004)
Region FE^f^	Yes	Yes	Yes	Yes
Weekday FE	Yes	Yes	Yes	Yes
Hour FE	Yes	Yes	Yes	Yes
Region × weekday × hour FE	Yes	Yes	Yes	Yes
Observations	236,775	236,775	236,775	236,775
*R* ^2^	0.804	0.804	0.804	0.804
Adjusted *R*^2^	0.804	0.804	0.804	0.804

^a^*TCN* denotes the total cumulative number of WEAs in a specific district since January 1, 2020, the beginning of the observation period.

^b^*TCC* denotes the total number of confirmed cases of COVID-19.

^c^*N*: number of WEAs.

^d^*DCN* denotes the daily cumulative number of WEAs in a specific district.

^e^Not applicable.

^f^FE: fixed effect.

## Discussion

### Principal Results

This study empirically measured the association between the details of issuing WEAs and the granular region-hour-level estimated floating population changes in 25 districts in Seoul, South Korea. We used variations in the number and content of WEAs independently managed by local authorities to empirically measure the WEAs’ impact. By estimating a panel-data model with a series of fixed effects, we found robust evidence that issuing WEAs was related to the reduction of the floating population in districts of Seoul. A collection of robustness and falsification tests showed that our findings were robust. Furthermore, we found that location was the only significant type of information among various types extracted from WEAs that was associated with a decrease in the floating population.

The information necessary for disaster response must be delivered promptly and accurately to overcome disaster situations. WEAs, which deliver disaster information directly to the recipients’ mobile phones, have become a major means of providing disaster information. One of the most efficient ways to respond to a disaster situation is to evacuate people from dangerous areas to safe locations. Therefore, a major objective of issuing WEAs is to control or reduce the floating population in hazardous areas to an appropriate level. These policy objectives gained further prominence during the COVID-19 pandemic, as the primary content of WEAs at the peak of the pandemic emphasized practicing social distancing and avoiding densely populated areas. Hence, an intuitive approach to measuring the impact of WEAs is to examine the relationship between the number of WEAs sent and the size of the floating population in a given area. Our empirical data set was ideal for this study due to the following reasons: (1) The data set provided a highly informative empirical setting to measure the impact of WEAs, as different local governments sent WEAs with various region-hour-level data in districts of Seoul, which are identifiable; (2) The data set contained granular information on floating populations with various region-hour–level data, which were compatible with the WEA data set; (3) WEAs are mandatory for mobile users; and lastly, (4) the empirical data set fully covered the COVID-19 pandemic in South Korea after the first confirmed case was reported, which provides us with a clear impact in a long-enough range. Overall, our empirical data were ideal for studying our research question.

This study makes several contributions. First, this study offers new empirical evidence on the impact of WEAs, advancing previous research that mainly relied on hypothetical scenario surveys due to limited WEAs and challenges in identifying key variables [10-12]. Earlier studies, particularly those examining COVID-19, faced difficulties in robust statistical analysis and data collection reflecting public responses [3,13]. Unlike previous work that focused on a few regions or solely on the number of WEAs, this study stands out by analyzing variations in the number and content of WEAs across different regions due to the regions’ independent authorities issuing them [8].

Second, this study provides a plausible mechanism for how WEAs can reduce other focal outcomes, such as the numbers of infections and deaths [3], which remain unclear. Previous studies revealed that population density has a strong correlation with the number of infections and deaths from COVID-19 [14-17]. Transmitting WEAs reduces the floating population in the area where WEAs are sent and consequently reduces the confirmed COVID-19 cases and deaths.

Lastly, this study provides important implications for developing a policy on making optimal WEAs to prevent national disasters or pandemics, which suggests that location information should be highlighted rather than other information (eg, date and general information).

### Limitations

This study has some limitations due to technical issues with the WEA system and the difficulty in obtaining data sets. This study observed the relationship between the transmission history of WEAs in districts and the change in the floating population. However, we could not confirm whether the people who received WEAs moved to a safe place, which makes it hard to argue for a causal relationship. To supplement this limitation, we require an additional data set that can check the WEA confirmation time and movement history through personal-level mobile phone log records.

We used the estimated floating population provided by SK Telecom as a dependent variable. However, it is worth noting that while SK Telecom has the largest number of subscribers, with a market share of 44.3% as of 2021 [18], the floating population they estimated and provided may have differed from the actual floating population. We conducted an additional analysis using the floating population provided by the Seoul local government as the dependent variable, and the results are summarized in Multimedia Appendix 2. The implications of this study remained consistent even when analyzed with an additional data set.

This study focused on WEAs and the floating population of 25 districts in Seoul, the capital and largest city of South Korea. For a more generalized conclusion about the effectiveness of WEAs, it is necessary to expand and analyze the data set to include more regions in future studies.

WEAs can be blocked by changing mobile phone settings; in that case, the impact of transmitting WEAs on behavioral responses may vary. Nevertheless, due to technical constraints, the government cannot collect data on the rate of WEA blocking. The WEA sending system should be developed so that the WEA reception or blocking rate can be aggregated and used for policy formulation.

### Conclusions

This study analyzed the impact of WEAs sent in the 25 districts of Seoul on changes in the floating population in those areas, using the fact that the content of the WEAs sent during the COVID-19 pandemic was focused on compliance with social distancing guidelines. The results showed that issuing WEAs and distributing information related to COVID-19 to a specific district in Seoul, South Korea, was related to the reduction of the floating population of that district. Furthermore, among the 4 types of information (location, date, order, and disease) in the WEAs, location information was the only significant type that was associated with a decrease in the floating population. Contrasting with previous studies that could not generalize findings by using hypothetical survey results or analyzing WEAs and the floating population in a single region, this study empirically elucidates the impact of WEAs on public behavior by examining the number and content of region-hour–level messages sent by 25 districts with independent dispatch authority, along with the corresponding changes in the floating population.

Our findings provided a plausible mechanism by which WEAs could effectively suppress infectious diseases by providing clues that transmitting WEAs was related to the reduction of the floating population during the COVID-19 pandemic. Our findings focused on a correlational relationship rather than a causal one, as we were unable to observe whether people who received WEAs moved to safer places.

We hope that this research will spur further investigation into the sophisticated measurement of WEA impact and the establishment of disaster response systems.

## Appendix

Multimedia Appendix 1 Summary of the number of wireless emergency alerts across districts (Table S1) and monthly number of wireless emergency alerts across districts (Table S2).

Multimedia Appendix 2 Additional analyses with a new data set.

## Abbreviations

API: application programming interface
CBS: cell broadcast service
NER: named entity recognition
WEA: wireless emergency alert
