# Supplementary material for: The Impact of Wireless Emergency Alerts on a Floating Population in Seoul, South Korea: Panel Data Analysis
Source: JMIR Public Health Surveill. 2024 Mar 25;10:e43554. doi: 10.2196/43554 (PMC10964982; doi:10.2196/43554)
Supplement: Multimedia Appendix 1 [file publichealth_v10i1e43554_app1.docx]

## Multimedia Appendix 1

We summarize the number of WEAs across districts in Table S1 and present the monthly number of WEAs across districts in Table S2.

**Table S1**. Total number of wireless emergency alerts across 25 districts in Seoul, January 2020-January 2021.

| District | N | % | District | N | % |
| --- | --- | --- | --- | --- | --- |
| Dongjak | 496 | 7.7 | Guro | 226 | 3.5 |
| Jungnang | 461 | 7.2 | Dongdaemun | 224 | 3.5 |
| Dobong | 395 | 6.1 | Yangcheon | 222 | 3.4 |
| Gangnam | 355 | 5.5 | Yeongdeungpo | 211 | 3.3 |
| Gwanak | 327 | 5.1 | Jongno | 208 | 3.2 |
| Seocho | 290 | 4.5 | Geumcheon | 198 | 3.1 |
| Gangseo | 286 | 4.4 | Seongbuk | 197 | 3.1 |
| Songpa | 269 | 4.2 | Seongdong | 187 | 2.9 |
| Gwangjin | 261 | 4.0 | Seodaemun | 183 | 2.8 |
| Gangdong | 257 | 4.0 | Yongsan | 180 | 2.8 |
| Nowon | 253 | 4.0 | Mapo | 179 | 2.888 |
| Gangbuk | 233 | 3.6 | Jung | 114 | 1.8 |
| Eunpyeong | 230 | 3.6 | Total | 6,442 | 100 |

**Table S2**. Monthly number of wireless emergency alerts across 25 districts in Seoul, January 2020-January. 2021.

| **District** | **Jan. 20** | **Feb. 20** | **Mar. 20** | **Apr. 20** | **May 20** | **Jun. 20** | **Jul. 20** | **Aug. 20** | **Sep. 20** | **Oct. 20** | **Nov. 20** | **Dec. 20** | **Jan. 21** |
| --- | --- | --- | --- | --- | --- | --- | --- | --- | --- | --- | --- | --- | --- |
| Gangnam | 2 | 2 | 14 | 61 | 22 | 22 | 30 | 42 | 32 | 25 | 29 | 31 | 43 |
| Gangdong |  | 3 | 6 | 3 | 10 | 11 | 8 | 35 | 27 | 18 | 39 | 51 | 46 |
| Gangbuk |  |  | 14 |  | 13 | 10 | 6 | 34 | 25 | 14 | 20 | 47 | 50 |
| Gangseo |  | 2 | 14 | 5 | 21 | 33 | 14 | 41 | 36 | 14 | 37 | 33 | 36 |
| Gwanak |  | 4 | 31 | 13 | 20 | 40 | 27 | 40 | 31 | 27 | 23 | 37 | 34 |
| Gwangjin | 1 | 1 | 11 | 6 | 12 | 8 | 17 | 66 | 34 | 12 | 26 | 34 | 33 |
| Guro |  |  | 31 | 2 | 11 | 23 | 5 | 22 | 24 | 14 | 22 | 39 | 33 |
| Geumcheon |  | 2 | 23 | 3 | 6 | 14 | 7 | 22 | 19 | 7 | 17 | 50 | 28 |
| Nowon |  | 2 | 11 |  | 2 | 1 |  | 35 | 33 | 21 | 29 | 57 | 62 |
| Dobong |  | 2 | 15 | 5 | 18 | 37 | 12 | 69 | 56 | 29 | 40 | 50 | 62 |
| Dongdaemun |  | 3 | 25 | 5 | 3 | 4 | 6 | 38 | 22 | 16 | 36 | 34 | 31 |
| Dongjak |  | 3 | 25 | 12 | 27 | 23 | 30 | 99 | 73 | 27 | 53 | 74 | 50 |
| Mapo |  | 2 | 13 | 5 | 6 | 10 | 9 | 27 | 16 | 13 | 17 | 31 | 30 |
| Seodaemun |  | 1 | 7 | 6 | 10 | 5 | 6 | 27 | 24 | 8 | 27 | 31 | 31 |
| Seocho |  | 1 | 3 | 30 | 21 | 20 | 15 | 43 | 20 | 18 | 44 | 40 | 35 |
| Seongdong |  |  | 7 | 10 | 17 | 8 | 6 | 19 | 17 | 13 | 22 | 34 | 34 |
| Seongbuk |  | 1 | 14 | 7 | 4 | 5 | 9 | 28 | 22 | 10 | 27 | 36 | 34 |
| Songpa | 1 | 7 | 16 | 9 | 11 | 11 | 18 | 36 | 27 | 20 | 37 | 32 | 44 |
| Yangcheon |  | 2 | 14 | 4 | 9 | 29 | 1 | 40 | 21 | 10 | 25 | 32 | 35 |
| Yeongdeungpo |  | 2 | 16 | 9 | 16 | 17 | 6 | 25 | 27 | 8 | 24 | 30 | 31 |
| Yongsan |  |  | 10 | 8 | 11 | 9 | 4 | 23 | 14 | 14 | 26 | 31 | 30 |
| Eunpyeong |  | 4 | 14 | 8 | 8 | 13 | 8 | 40 | 30 | 15 | 26 | 33 | 31 |
| Jongno |  |  | 6 | 3 | 14 | 20 | 21 | 29 | 22 | 13 | 22 | 32 | 26 |
| Jung | 1 |  | 1 |  | 4 |  | 2 | 8 | 7 | 5 | 21 | 33 | 32 |
| Jungnang |  | 1 | 19 | 3 | 3 | 15 | 14 | 71 | 46 | 37 | 52 | 116 | 84 |
| Total | 5 | 45 | 360 | 217 | 299 | 388 | 281 | 959 | 705 | 408 | 741 | 1048 | 985 |
| Average | 1.3 | 2.4 | 14.4 | 9.9 | 12.0 | 16.2 | 11.7 | 38.4 | 28.2 | 16.3 | 29.6 | 41.9 | 39.4 |

Figure S1 depicts the monthly floating population means across 25 districts. The x-axis represents the time at the month level, whereas the y-axis represents the mean of the floating population across 25 districts. The line with dots represents the time trend. As illustrated in Figure S1, the floating population decreases over time.

**Figure S1**. Monthly mean of the floating population across 25 districts in Seoul, January 2020-January 2021.
