# Supplementary material for: The Impact of Wireless Emergency Alerts on a Floating Population in Seoul, South Korea: Panel Data Analysis
Source: JMIR Public Health Surveill. 2024 Mar 25;10:e43554. doi: 10.2196/43554 (PMC10964982; doi:10.2196/43554)
Supplement: Multimedia Appendix 2 [file publichealth_v10i1e43554_app2.docx]

## Multimedia Appendix 2

In this section, we have conducted additional analyses with a new dataset. The new dataset provided by Seoul local government contains the floating population information across districts at the hour level in Seoul likewise the dependent variable for the main analysis. The details of the data generation process are not publicly available, but it indicates the number of people who stayed in a certain area of Seoul at a given time, measured by combining the administrative data of Seoul and the location-based population counts provided by domestic mobile carriers. We re-run the main specifications to check whether our findings hold with a different dataset.

Table S1 and Table S2 summarize the descriptive statistics of the dependent variables, across month and region. The floating populations from the two different datasets have a very high correlation coefficient of about 0.95, indicating a fairly similar flow. This means that the floating population used in the main body of this study is representatively inferring the flow of the floating population in Seoul, although it is derived from subscribers of a single carrier provider.

**Table S1**. Monthly descriptive statistics of the floating population.

| Source | SK Telecom | | Seoul | |
| --- | --- | --- | --- | --- |
|  | Mean | Std.dev | Mean | Std.dev |
| **Jan.20** | 329,549 | 109,684 | 429,179 | 153,121 |
| **Feb.20** | 339,421 | 124,847 | 434,419 | 153,647 |
| **Mar.20** | 324,244 | 103,571 | 428,188 | 148,254 |
| **Apr.20** | 327,213 | 104,553 | 426,606 | 148,027 |
| **May.20** | 325,598 | 104,807 | 422,965 | 146,762 |
| **Jun.20** | 327,726 | 107,395 | 423,997 | 148,498 |
| **Jul.20** | 327,292 | 108,489 | 423,312 | 150,244 |
| **Aug.20** | 321,018 | 103,179 | 415,892 | 145,063 |
| **Sep.20** | 323,601 | 102,355 | 417,807 | 143,127 |
| **Oct.20** | 324,009 | 104,844 | 415,250 | 144,417 |
| **Nov.20** | 327,389 | 106,504 | 417,979 | 146,330 |
| **Dec.20** | 326,026 | 102,798 | 414,427 | 141,819 |
| **Jan.21** | 333,612 | 106,549 | 414,870 | 144,019 |

**Table S2**. Total number of wireless emergency alerts across districts.

|  | SK Telecom | | Seoul | |
| --- | --- | --- | --- | --- |
|  | Mean | Std.dev | Mean | Std.dev |
| Gangnam | 584,296 | 154,871 | 802,227 | 162,270 |
| Gangdong | 331,660 | 29,472 | 504,524 | 28,207 |
| Gangbuk | 237,112 | 25,009 | 303,665 | 21,001 |
| Gangseo | 441,960 | 28,718 | 542,031 | 18,975 |
| Gwanak | 393,731 | 46,970 | 486,869 | 40,511 |
| Gwangjin | 283,634 | 25,163 | 366,784 | 17,952 |
| Guro | 355,082 | 21,542 | 389,295 | 10,138 |
| Geumcheon | 226,477 | 30,116 | 223,527 | 23,948 |
| Nowon | 396,321 | 42,786 | 526,378 | 31,623 |
| Dobong | 240,607 | 28,856 | 281,833 | 17,819 |
| Dongdaemun | 290,070 | 16,429 | 354,573 | 8,528 |
| Dongjak | 310,161 | 27,853 | 394,352 | 25,293 |
| Mapo | 338,553 | 29,958 | 452,297 | 29,734 |
| Seodaemun | 251,643 | 14,431 | 359,450 | 15,113 |
| Seocho | 407,764 | 80,850 | 568,985 | 87,309 |
| Seongdong | 256,085 | 21,902 | 342,247 | 17,878 |
| Seongbuk | 337,422 | 30,341 | 427,263 | 22,750 |
| Songpa | 516,910 | 28,518 | 747,868 | 20,659 |
| Yangcheon | 326,712 | 34,078 | 398,938 | 22,217 |
| Yeongdeungpo | 390,838 | 58,478 | 455,906 | 56,565 |
| Yongsan | 210,650 | 22,455 | 282,847 | 23,562 |
| Eunpyeong | 335,388 | 44,105 | 449,731 | 38,540 |
| Jongno | 211,802 | 76,398 | 273,717 | 70,915 |
| Jung | 215,151 | 108,352 | 250,776 | 95,854 |
| Jungnang | 294,515 | 34,092 | 360,286 | 26,937 |

We conducted several robustness checks. First, we used subsamples where the observations are in the early stages to minimize the possibility that people may stop receiving WEAs. The results are summarized in Table S3. According to the “Naver trend”, a service that shows the search trend of words searched by users on “Naver”, the largest engine service provider in Korea, we sample the observations within the first 82 days, until March 23, 2020, when the search volume for how to turn off WEAs was the highest. As shown in Table S3, the results with subsamples are consistent with the main findings in Table 3 that issuing WEAs are associated with a decrease in the floating population, regardless of the specifications.

**Table S3**. Result from a subsample of the first 82 days of data set: Panel regression model using wireless emergency alerts (WEAs) and the floating population at the district-hour level with a series of fixed effects.

|  | (1) | (2) | (3) | (4) |
| --- | --- | --- | --- | --- |
|  | Base | With *TCN*^b^ | With *TCC*^c^ | With time |
| N | −0.033 | −0.025 | −0.025 | −0.031 |
|  | (0.011; P=.005) | (0.010; P=.02) | (0.010; P=.02) | (0.010; P=.006) |
| *DCN*^a^ | −0.013 | −0.0043 | −0.0056 | −0.011 |
|  | (0.0027; P<.001) | (0.0026; P=.11) | (0.0029; P=.07) | (0.0027; P<.001) |
| Log (*TCN*)^b^ |  | −0.0087 |  |  |
|  |  | (0.0011; P<.001) |  |  |
| Log (*TCC*)^c^ |  |  | −0.0088 |  |
|  |  |  | (0.0016; P<.001) |  |
| Log (*Day*) |  |  |  | −0.0039 |
|  |  |  |  | (0.0012; P=.003) |
| Region FE | Yes | Yes | Yes | Yes |
| Weekday FE | Yes | Yes | Yes | Yes |
| Hour FE | Yes | Yes | Yes | Yes |
| Region × weekday × hour FE | Yes | Yes | Yes | Yes |
| Observations | 49,200 | 49,200 | 49,200 | 49,200 |
| R-squared | 0.755 | 0.755 | 0.755 | 0.755 |
| Adjusted R-squared | 0.752 | 0.752 | 0.752 | 0.752 |

^a^ *DCN* denotes the daily cumulative number of WEAs in a specific district.

^b^ *TCN* denotes the total cumulative number of WEAs in a specific district since January 1, 2020, the beginning of the observation period.

^c^ *TCC* denotes the total number of confirmed cases of COVID-19.

Second, we add several lagged WEAs to the main specification. The results are consistent regardless of the lagged variables, as shown in column (1) of Table S4. Meanwhile, in column (2) of Table S4, we use information extracted from WEAs to investigate which type of information might lead to a decrease in the floating population. Four types of information, which are included in numerous WEAs and may contain key information related to the pandemic situation, were identified and analyzed in the text of WEAs: location, date, order, and disease. The results in column (2) of Table S4 suggest that location information is associated with a decrease in the floating population. According to the estimation result, one increase in WEA that includes location information is related to a reduction of the floating population by 1.2% ($=100(1-e^{-0.012})$).

**Table S4**. Results including Lagged wireless emergency alerts (WEAs) and WEA information: Panel regression model using WEAs and the floating population at the district-hour level with a series of fixed effects.

|  | (1) | (2) |
| --- | --- | --- |
|  | Lagged WEAs | WEA information |
| N | −0.015 |  |
|  | (0.0048; P=.005) |  |
| *DCN*^a^ | −0.0057 | −0.0035 |
|  | (0.0037; P=.13) | (0.0036; P=.34) |
| *Log (TCN)*^b^ | −0.0035 | −0.0019 |
|  | (0.0006; P<.001) | (0.0006; P=.002) |
| Lag 1 | −0.0002 |  |
|  | (0.0008; P=.75) |  |
| Lag 2 | 0.0017 |  |
|  | (0.0008; P=.06) |  |
| Lag 3 | 0.0009 |  |
|  | (0.0006; P=.11) |  |
| Lag 4 | −0.0001 |  |
|  | (0.0010; P=.91) |  |
| Lag 5 | 0.0029 |  |
|  | (0.0012; P=.02) |  |
| Lag 6 | 0.0012 |  |
|  | (0.0008; P=.15) |  |
| Lag 7 | 0.0017 |  |
|  | (0.0014; P=.23) |  |
| Location |  | −0.012 |
|  |  | (0.0036; P=.003) |
| Date |  | 0.0087 |
|  |  | (0.0099; P=.39) |
| Order |  | −0.0062 |
|  |  | (0.0042; P=.15) |
| Disease |  | 0.0061 |
|  |  | (0.0075; P=.42) |
| Region FE | Yes | Yes |
| Weekday FE | Yes | Yes |
| Hour FE | Yes | Yes |
| Region × weekday × hour FE | Yes | Yes |
| Observations | 236,775 | 236,775 |
| R-squared | 0.804 | 0.804 |
| Adjusted R-squared | 0.804 | 0.804 |

^a^ *DCN* denotes the daily cumulative number of WEAs in specific district.

^b^ *TCN* denotes the total cumulative number of WEAs in specific district since January 1, 2020, the beginning of the observation period.

We perform falsification tests to demonstrate that our main findings do not hold when using incorrect variables in terms of district and timing. The first falsification is the use of variables in the “incorrect” district. We designated an incorrect district as one of the districts that differed from the true district. As shown in column (1) of Table S5, the coefficient of *N* is no longer significant. The second falsification is the use of “incorrect” time. We set an incorrect hour to one of the hours different from the true hour. For instance, the number of WEAs at noon can be replaced by the number of WEAs at 9 PM. The coefficient of *N* is again no longer significant, as shown in column (2) of Table S5.

**Table S5**. Parameter estimates of the main empirical model using an additional data set.

|  | (1) | (2) |
| --- | --- | --- |
|  | Falsification:  incorrect region | Falsification:  incorrect time |
| N | 0.0019 | 0.0018 |
|  | (0.0053;  P=.72) | (0.0040;  P=.65) |
| *DCN*^a^ | 0.0052 | 0.0027 |
|  | (0.0033; P=.13) | (0.0020;  P=.18) |
| *Log (TCN)*^b^ | −0.0029 | −0.0025 |
|  | (0.0008; P<.001) | (0.0005; P<.001) |
| Region FE | Yes | Yes |
| Weekday FE | Yes | Yes |
| Hour FE | Yes | Yes |
| Region × weekday × hour FE | Yes | Yes |
| Observations | 236,775 | 236,775 |
| R-squared | 0.804 | 0.804 |
| Adjusted R-squared | 0.804 | 0.804 |

We have summarized the results of main specifications with a new dataset in Table S6. We include district, weekday, hour, and joint district-hour fixed effects in all regressions likewise the main analysis.

**Table S6**. Parameter estimates of the main empirical model using an additional data set.

|  | (1) | (2) | (3) | (4) |
| --- | --- | --- | --- | --- |
|  | Base | With TCN | With TCC | With Day |
| *N* | −0.017 | −0.010 | −0.010 | −0.011 |
|  | (0.0044; P<.001) | (0.0040; P=.01) | (0.0039; P=.02) | (0.0040; P=.009) |
| *DCN* | −0.0099 | −0.0033 | −0.0029 | −0.0044 |
|  | (0.0026; P=.001) | (0.0026; P=.21) | (0.0025; P=.25) | (0.0025; P=.099) |
| *Log (TCN)* |  | −0.0067 |  |  |
|  |  | (0.0006; P<.001) |  |  |
| *Log (TCC)* |  |  | −0.0063 |  |
|  |  |  | (0.0006; P<.001) |  |
| *Log (Day)* |  |  |  | −0.0116 |
|  |  |  |  | (0.0010; P<.001) |
| Region FE | Yes | Yes | Yes | Yes |
| Weekday FE | Yes | Yes | Yes | Yes |
| Hour FE | Yes | Yes | Yes | Yes |
| Region × weekday × hour FE | Yes | Yes | Yes | Yes |
| Observations | 236,775 | 236,775 | 236,775 | 236,775 |
| R-squared | 0.905 | 0.906 | 0.906 | 0.906 |
| Adjusted R-squared | 0.905 | 0.906 | 0.906 | 0.906 |

As shown in the first row of Table S6 (*N*), the estimates are significantly negative in all specifications, which is consistent with the main findings in Table 3. According to specification (2), one increase in issuing WEA was related to a decrease in the floating population by 1.0%. Other specifications also yield similar results, ranging from 1.0% to 1.7%. Although the coefficients of *DCN*, which represents the effect of daily cumulative WEAs, are also negative across all models, some estimates are insignificant. In specification (2), we include *TCN*, which indicates the cumulative number of WEAs from the beginning of the observation period. The results show that cumulative WEAs are also negatively correlated with the floating population. To check the robustness of the results, we replaced *TCN* with *TCC* (total number of confirmed cases of COVID-19) and *Day* (daily time trend) and summarized the estimates in columns (3) and (4). *TCC* and *Day* have negative impacts on the floating population like *TCN,* which is consistent with the main finding.
